# Identification of non-tuberculous mycobacteria isolated from clinical specimens at a tertiary care hospital: a cross-sectional study

**DOI:** 10.1186/1471-2334-13-493

**Published:** 2013-10-22

**Authors:** Imran Ahmed, Kauser Jabeen, Rumina Hasan

**Affiliations:** 1 Department of Pathology & Microbiology, Aga Khan University Hospital, Karachi, Pakistan

**Keywords:** Non-tuberculous mycobacteria, Identification, Conventional tests, Drug susceptibility testing, Pakistan

## Abstract

**Background:**

Non-tuberculous mycobacteria (NTM) are opportunistic pathogens in immuno-compromised patients. They are also increasingly recognized as pathogens in immuno-competent individuals. Globally, an increase in NTM isolation is being reported with a varied geographic prevalence of different species around the world. There is lack of data on species distribution of these organisms from Pakistan. Treatment options differ according to the species isolated and its susceptibility profile. Knowledge of local species variation would help targeted therapy. This study was performed to determine frequencies of different NTM species isolated from various clinical specimens submitted at a tertiary care hospital laboratory.

**Methods:**

NTM isolated from 25955 clinical specimens over a period of two years (2010 to 2011) were included. All NTM were identified using conventional tests. Drug susceptibility testing (DST) was performed by broth microdilution and interpreted according to Clinical and Laboratory Standards Institute’s document M24-A2.

**Results:**

A total of 104 NTM were included in the study. Of these, 76% (54/71) rapidly growing mycobacteria (RGM) and 57.6% (19/33) slow growing mycobacteria (SGM) could be further identified. *Mycobacterium fortuitum* (21/54) was the commonest NTM identified among RGM followed by *M. mucogenicum* (12/54) and *M. smegmatis* (11/54). Among SGM, *M. avium* complex (MAC) was the most frequent (14/19). Clinical significance could be assessed in a limited number (52/104) of NTM isolates and MAC appeared to be the commonest significant NTM. Three extra-pulmonary cases were found to be healthcare associated infections. DST results for RGM showed susceptibility to amikacin (100%), clarithromycin (100%, except *M. fortuitum* where it is not reportable), linezolid (90%) and moxifloxacin (75%). Whereas SGM were susceptible to clarithromycin (100%), linezolid (58.8%) and moxifloxacin (64.7%).

**Conclusion:**

This is the first study reporting NTM species and their clinical significance isolated from clinical specimens from Pakistan. Isolation of NTM from clinical specimens should prompt to evaluate their clinical significance.

## Background

Non-tuberculous mycobacteria (NTM) also known as mycobacteria other than tuberculosis (MOTT) are acid fast bacilli that do not belong to *Mycobacterium tuberculosis* complex or *Mycobacterium leprae*. These mycobacteria are environmental organisms and are found in natural bodies of waters, biofilms, soil, water damaged walls, etc. NTM may also be found in drinking water supplies. Humans may get infected with NTM from environmental sources but unlike *M. tuberculosis* and *M. leprae*, evidence for human to human transmission is lacking. NTM are globally recognized as pathogens especially in immuno-compromised population including HIV/AIDS (Human Immunodeficiency Virus/Acquired Immunodeficiency Syndrome) patients [1,2]. In addition to causing infections in immuno-compromised patients, this group of organisms has been reported to be increasingly recognized as pathogens from immuno-competent individuals as well [3,4].

There has been an increase in the rate of isolation of NTM. A multi-country retrospective study reported a diverse and increasing trend of NTM isolation in 14 countries of the world over a period spanning several years [5]. This study also highlighted geographical variation in the pattern of NTM isolation across participating countries. For example, *M. fortuitum* was the commonest species reported from Iran and Turkey whereas *M. avium* complex (MAC) predominated from most of the European countries and Brazil. From Belgium, *Mycobacterium xenopi* while *M. gordonae* was the most frequently isolated NTM from Czech Republic. An increase in the number of isolation of NTM may be explained by a number of reasons including increase in the number of immuno-compromised population including HIV/AIDS, introduction of new technologies to dissect out related NTM species, increasing knowledge and interest in the isolation of all *Mycobacterium* species and impact of human activities on the ecology of NTM [6]. An increasing trend and geographical variation in NTM species isolation is also reported from India [7] and Taiwan [8].

Rising number of NTM isolation is of concern as these are both difficult to diagnose and treat and the treatment also varies according to the NTM species involved, its susceptibility profile and the disease site [9]. Treatment of these infections usually involves a combination of drugs with *in vitro* activity against a particular NTM isolate. Various methods including agar disk elution, agar disk diffusion, E-test have been described but broth microdilution is considered to be the gold standard for performing drug susceptibility testing (DST) of NTM. Interpretation criteria are best defined for MAC, *M. kansasii*, *M. marinum* and rapidly growing mycobacteria (RGM) until more data become available for other NTM species [10]. A recently published review on drug susceptibility testing, resistance mechanisms and therapy of NTM infections further highlights the importance of recognizing NTM species and performing DST on significant clinical isolates [11].

Pakistan is a high burden country for tuberculosis [12] and patients with chronic pneumonia, meningitis, lymphadenitis, pyrexia of unknown origin, chronic non-healing ulcers/wounds and other chronic infections are evaluated for tuberculosis by performing microbiological cultures of various clinical specimens. Besides isolation of *M. tuberculosis*, NTM are also isolated from these specimens. Most of the clinical microbiology laboratories in Pakistan differentiate *Mycobacterium* species into *M. tuberculosis* and non-tuberculous mycobacteria and do not attempt to identify NTM to species level. NTM isolated from clinical specimens are reported without further identification. From Pakistan, information is lacking regarding NTM species isolated from clinical specimens. Therefore, there is a need to identify NTM to species level that would help targeted treatment.

## Methods

### Bacterial strains

NTM were prospectively collected from Clinical Laboratory, Aga Khan University Hospital (AKUH), Karachi, Pakistan, from 2010 to 2011. During the study period, 104 NTM were isolated. Strains were saved at -80°C and revived when required. The hospital and its clinical laboratory are accredited by the Joint Commission International Accreditation (JCIA).

Assessment of the clinical significance of an NTM isolate is vital for establishing its role in causing patient’s symptoms. Clinical information is routinely collected at AKUH Clinical Microbiology Laboratory as good clinical practice to serve this purpose. This included presenting symptoms, occupation, co-morbids (e.g. malignancy, HIV infection), smoking, previous tuberculosis or tuberculosis treatment history, chronic obstructive pulmonary disease, cystic fibrosis, immunosuppression (HIV, malignancy, chemotherapy, steroid intake), surgical procedure (as a risk factor for extra-pulmonary NTM infections) and chest X-ray and/or CT scan findings. This information has been used and presented in this study anonymously. Significance assessment of NTM isolation was carried out in cases where clinical information was available. For extra-pulmonary NTM isolates, diagnosis was assessed on patient history and by isolation of NTM from aspirated pus, tissue biopsies or sterile body fluids. Diagnosis of pulmonary NTM disease was made by criteria (where available) published by American Thoracic Society/Infectious Diseases Society of America which combines clinical and microbiological criteria: 1. Clinical symptoms & appropriate exclusion of other diseases, and 2. Microbiological (positive culture from at least two sputum samples, or positive culture from one bronchial wash or lavage) [9].

Cultures were performed using Lowenstein Jensen (LJ), Mycobacterium Growth Indicator Tube (MGIT) and Middlebrook 7H10 agar for all the specimens by standard microbiological procedures [13].

### Identification of NTM

Colonies growing on solid media were confirmed as AFB by performing Kinyoun stain. Growth rate (as determined by subculturing the organism onto Middlebrook 7H10 agar after growing a well isolated colony in Middlebrook 7H9 broth with Tween-80) and colony pigmentation were assessed. Para-nitro benzoic acid (PNB) sensitive and thiophene-2-carboxylic acid hydrazide (T2H) resistant strains (*M. tuberculosis* ssp. bovis is T2H sensitive) were identified as *M. tuberculosis* complex and excluded from the study. NTM were further identified by following conventional tests: rate of growth, pigment production, arylsulfatase, heat-stable catalase, growth on MacConkey agar without crystal violet, iron uptake, nitrate reduction, tween-80 hydrolysis, tellurite reduction and urease production [13]. American Type Culture Collection (ATCC) strains (*M. fortuitum* ATCC6841, *M. avium* ATCC 25291, *M. tuberculosis* ATCC 15177, *M. kansasii* TCC12478, *M. phlei* ATCC11758) and/or selected strains received from College of American Pathologists’ survey were included as positive and negative controls (if ATCC strains were not available) for all the tests performed for the identification of NTM.

### Susceptibility testing

Broth microdilution was used to perform drug susceptibilities using 96 well sensititre plates (TREK Diagnostic Systems Ltd, UK) as per manufacturer’s recommendations. Susceptibility data thus obtained was interpreted according to the Clinical and Laboratory Standards Institute’s criteria (Susceptibility testing of Mycobacteria, Nocardia and other aerobic Actinomycetes: Approved standard—second edition. CLSI document M24-A2. 2011) [10].

## Results

**Figure 1:**
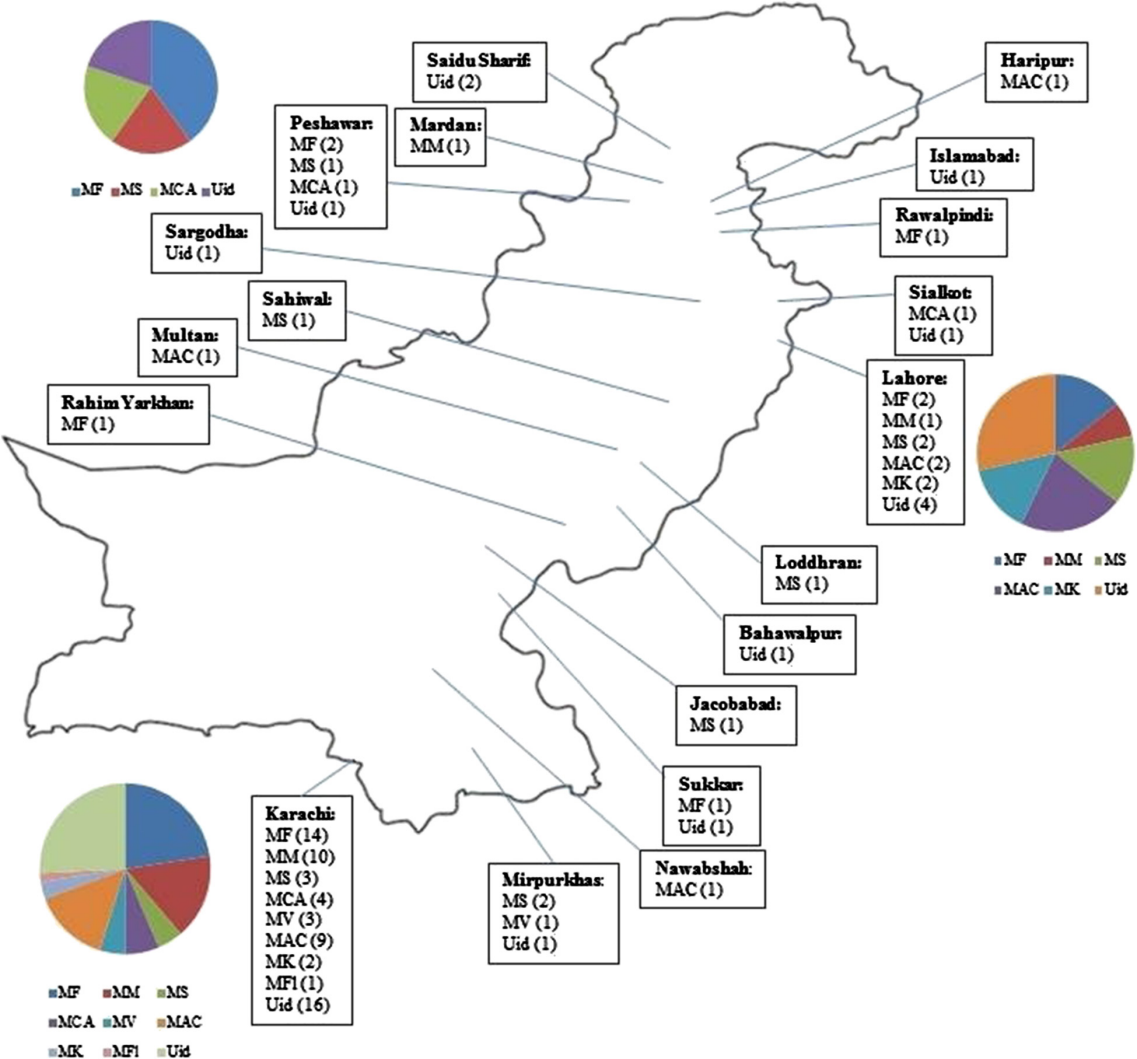
**Map of Pakistan showing geographical location of various NTM isolated.** Pie charts represnet spectrum of NTM isolated: bottom left (NTM isolated in Karachi); top left (NTM isolated in Peshawar); right (NTM isolated in Lahore). Name of cities are given in bold in each box, numbers in brackets represent number of each NTM isolated in that city. MF, *M. fortuitum*; *M. mucogenicum*; MS, *M. smegmatis*; MCA, *M. chelonae-abscessus* group; MV, *M. vaccae*; MAC, *M. avium* complex; MK, *M. kansasii*; MFl, *M. flavescens*; Uid, unidentified NTM.

A total of 104 clinical NTM isolates were included in the study. Of these, 92 NTM were isolated from pulmonary specimens (sputum, tracheal aspirate and bronchoalveolar lavage fluid) and 12 from extrapulmonary specimens (pus, tissue biopsies, synovial fluid, pleural fluid) (Table 1). Seventy one (71/104, 68%) NTM were rapidly growing mycobacteria (RGM) and 33 (33/104, 32%) were slow growing mycobacteria (SGM). Among RGM, 54/71 (76%) and 19/33 (57.6%) SGM could be further identified. Most of the RGM were *M. fortuitum* (21/54, 39%) followed by *M. mucogenicum* (12/54, 22%) and *M. smegmatis* (11/54, 20%). Among SGM, most common species was found to be *M. avium* (14/19, 73.7%). A map of Pakistan showing geographical location of various NTM isolated in our study is shown in Figure 1.

**Table 1:** Distribution of non-tuberculous mycobacteria (NTM) from clinical specimens

	**NTM identified**	**Pulmonary isolates**	**Extra-pulmonary isolates**	**Total**
**RGM**				
	*M. fortuitum*	16	5	21
	*M. chelonae-abscessus*	6	0	6
	*M. mucogenicum*	9	3	12
	*M. smegmatis*	11	0	11
	*M. vaccae*	4	0	4
	Unidentified	17	0	17
**SGM**				
	*M. avium* complex	11	3	14
	*M. kansasii*	4	0	4
	*M. flavescens*	1	0	1
	Unidentified	13	1	14
Total		92	12	104

*RGM* = rapidly growing Mycobacteria; *SGM* = slowly growing mycobacteria.

Clinical information was available for 50% of the isolates (52 NTM from 42 patients) to assess significance of NTM isolated. Table 2 provides details of pulmonary and extrapulmonary clinically significant NTM isolates. The reason for including some of the patients with only one sputum culture positive for NTM was based on a strong clinical suspicion of NTM as the cause of patient’s symptoms and that most of these patients had AFB smear-positive from the sample (2^nd^ sputum specimen could not be collected). Therefore, the diagnosis in these cases is not certain, but a potential one. Of the pulmonary isolates, MAC was found to be significant in 4 cases. *M. fortuitum*, *M. mucogenicum* and *M. smegmatis* were significant in one case each. For 5 cases, NTM could not be identified beyond SGM. For extra-pulmonary significant isolates, one case each was caused by *M. mucogenicum*, *M. chelonae-abscessus* group and *M. fortuitum*. All three of these cases appeared to be healthcare associated infections. MAC was isolated from 2 significant extra-pulmonary cases.

**Table 2:** Clinically significant NTM (pulmonary and extra-pulmonary) isolates and their distribution among patients according to specimen type

**Specimen/number of specimens**	**Specimen AFB smear**	**NTM isolated**
**Pulmonary isolates**		
Sputum/2	Numerous AFB	*M. fortuitum*
Sputum/1	Moderate AFB	*M. mucogenicum*
Sputum/2	Few AFB	*M. smegmatis*
Sputum/4	Numerous AFB	MAC
Sputum/1	Numerous AFB	SGM
Sputum/1	Numerous AFB	SGM
Sputum/1	Numerous AFB	MAC
Sputum/1	Negative	SGM
BAL/1	Numerous AFB	SGM
Sputum/1	Few AFB	SGM
Sputum/2	Few AFB	MAC
Sputum/1	Negative	MAC
**Extra-pulmonary isolates**		
Pus/2	Negative	*M. mucogenicum*
Pus/2	Negative	*M. chelonae/abscessus*
Synovial fluid, synovium & granulation tissue/3	Negative	*M. fortuitum*
Lymph node pus/1	Numerous AFB	MAC
Pus/1	Negative	MAC

*NTM*, non-tuberculous mycobacteria; *MAC*, *Mycobacterium avium* complex; *AFB*, acid fast bacilli; *BAL*, bronchoalveolar lavage; *SGM*, slow growing mycobacteria.

Table 3 describes drug susceptibility results for NTM. All RGM were found to be susceptible to amikacin and all *M. chelonae-abscessus* and unidentified RGM were susceptible to clarithromycin. Most of the RGM were also susceptible to linezolid (18/20, 90%) and moxifloxacin (15/20, 75%). Susceptibility to doxycycline and imipenem was noted in 25% and 20% of RGM isolates respectively. Among 20 RGM isolates 9 (45%) were cotrimoxazole susceptible. All SGM including MAC isolates were susceptible to clarithromycin. Whereas 4/6 (66.7%) MAC isolates were susceptible to linezolid and only one was susceptible to moxifloxacin. All *M. kansasii* isolates showed susceptibility to moxifloxacin.

**Table 3:** NTM species and antibiotic susceptibilities as determined by broth microdilution

**NTM spp.**	**n**	**Number and proportion (%) of susceptible strains to given antibiotics**							
		**AK**	**CIP**	**CLA**	**DOX**	**IPM**	**LZD**	**MOX**	**SXT**
*M. fortiutum*	6	6	4	-	1	1	5	4	3
*M. cheloane-abscessus*	6	6	4	6	1	3	5	6	4
Unidentified RGM	8	8	1	8	3	0	8	5	2
**Total RGM**	**20**	**20 (100)**	**9 (45)**	**14 (100)**	**5 (25)**	**4 (20)**	**18 (90)**	**15 (75)**	**9 (45)**
MAC	6	-	-	6	-	-	4	1	-
*M. kansasii*	4	-	1	4	-	-	3	4	2
Unidentified SGM	7	-	-	7	-	-	3	6	-
**Total SGM**	**17**	**-**	**-**	**17 (100)**	**-**	**-**	**10 (58.8)**	**11 (64.7)**	**-**

Lines labeled with Total RGM and Total SGM represent total number of RGM or SGM tested for each antibiotic listed.

NTM, non-tuberculous mycobacteria; *MAC*, *Mycobacterium avium* complex; n = number of isolates tested; *AK*, amikacin; *CIP*, ciprofloxacin; *CLA*, clarithromycin; *DOX*, doxycycline; *IPM*, imipenem; *LZD*, linezolid; *MOX*, moxifloxacin; *SXT*, cotrimoxazole.

## Discussion

Non-tuberculous Mycobacteria have gained much clinical significance in the last couple of decades not only in immuno-compromised but also in immuno-competent patients. Their ubiquitous distribution in nature and man-made ecologies put them at an advantage of having their hosts close to their ecological niches. NTM cause a wide variety of infections. Two broad categories include pulmonary and extra-pulmonary NTM infections. Therapy of these infections depends on the NTM species isolated, site of infection and its drug susceptibility profile. Current guidelines recommend speciation of all clinically significant NTM isolates [9]. Published literature describes geographical variation in NTM species distribution. Present study is the first from Pakistan describing identification of NTM species and their clinical significance from clinical specimens submitted to a tertiary care hospital.

A total of 104 NTM isolates were included in this study. Among these, 68% (71/104) were RGM and 32% (33/104) were SGM. This is in contrast to an Indian study where 87% of the isolates were reported to be RGM over a period of 6 years [7]. A study from Taiwan reported 41.4% of NTM as RGM isolated from 1997–2003 [8]. In this Taiwanese study 39% of the NTM were *Mycobacterium avium* complex (MAC). A study from Karachi, Pakistan, described *M. xenopi* as the most common while *M. fortuitum* as relatively rare NTM species among isolates collected from four laboratories in Karachi [14]. These results are in contrast with the present study and need further studies to confirm the findings reported.

In our study, 70% of the NTM could be identified (54/71 from RGM and 19/33 from SGM). *M. fortuitum* group (21/71, 29.6%) and MAC (14/33, 42.4%) were the most common RGM and SGM species isolated respectively in our study. From India, 46% and 41% of the NTM were *M. chelonae* and *M. fortuitum* respectively among all the species included in the study [7]. A multi-country study involving 14 countries [5], reported *M. fortuitum* as the commonest species from Iran and Turkey and MAC as most frequent isolate from European countries and Brazil. Belgium had the highest rate for *M. xenopi* followed by *M. gordonae*. Czech Republic was reported to have *M. gordonae* and *M. kansasii* as the most frequent NTM species isolated. These findings highlight local and regional differences in NTM species distribution.

Association of NTM isolate and clinical presentation is difficult particularly for respiratory isolates owing to the ubiquitous nature of these organisms. Multiple factors increase the probability of clinical significance of NTM: “recovery from multiple specimens or sites, recovery of the organism in large quantities (AFB smear-positive specimens) or recovery of an NTM isolate from a normally sterile site” [9]. A total of 27 isolates from 17 patients were found to be clinically significant. Table 2 describes distribution of NTM isolates according to specimen type in cases where clinical information was available. This could be performed for a proportion of cases because clinical details were available for a limited number of patients. Finding of MAC as the commonest species associated with clinical disease (4 pulmonary and 2 extra-pulmonary cases) followed by RGM is in accordance with a study from Taiwan where most cases were caused by MAC and RGM [8].

Healthcare associated NTM infections are well documented in the literature [15,16]. We were able to assess 3 such extra-pulmonary healthcare associated NTM infections. Medical instruments can easily become contaminated with NTM if these are washed with tap water containing NTM and/or failure to use mycobactericidal disinfectants [17]. Similarly, fluids used during surgical procedures or for irrigation of surgical wounds can become contaminated with NTM if adequate sterile practices are not adhered to, resulting in non-healing surgical wounds.

Apart from causing healthcare associated infections, NTM may also contaminate specimens during collection (e.g. specimen contamination by NTM contained in tap water used to rinse mouth before sputum collection) [18]. Similarly, laboratories may be reporting an increased number of NTM because of contamination of reagents used in processing of specimens [19]. Therefore, isolation of NTM from clinical specimens should be evaluated carefully in the light of clinical information to assess their significance.

Proper management of patients with a particular NTM infection depends on drug susceptibility testing performed by standard methodology. We performed DST of NTM isolates by broth microdilution which is a reference method as recommended by CLSI [10]. Because of limited information on pharmacokinetic data to inform decisions regarding therapeutic MICs, discrepancies exist between *in vitro* DST and *in vivo* treatment outcomes [20]. American Thoracic Society/Infectious Diseases Society of America (ATS/IDSA) guidelines generally recommend a combination of agents based on *in vitro* DST results. Amikacin was found to inhibit all our RGM isolates that were tested and similarly all RGM were susceptible to clarithromycin (except for *M. fortuitum* group where it is not recommended because of the presence of an inducible macrolide resistance '*erm*’ gene). Linezolid also seems to be a good option for RGM treatment. Quinolone susceptibility was found to be variable with 45% and 75% isolates susceptible to ciprofloxacin and moxifloxacin respectively suggesting that these agents may be used in treatment regimens. Imipenem results are not encouraging with only 20% RGM found susceptible. Therefore in our setting, treatment options for *M. fortuitum* infections would be a combination of two agents with *in vitro* activity and that may include amikacin with a quinolone, linezolid or cotrimoxazole or two oral agents if amikacin cannot be tolerated. Looking at the DST results, clarithromycin either with amikacin or linezolid appears to be an option for treating *M. chelonae-abscessus* infections. For MAC, all isolates were susceptible to clarithromycin and most (4/6, 66.7%) were susceptible to linezolid. Only one isolate was susceptible to moxifloxacin. Thus, clarithromycin which is the first line agent for the treatment of MAC infection in a multidrug regimen (including ethambutol, rifamycin and/or amikacin/streptomycin depending on the disease severity) appears a viable option. Though, rifampin, isoniazid, ethambutol, ethionamide, streptomycin and clarithromycin are inhibitory for *M. kansasii* isolates, but susceptibility breakpoints are not established for all of these drugs. Routine testing of rifampin and clarithromycin only is recommended for *M. kansasii* isolates. All our *M. kansasii* isolates were susceptible to clarithromycin and moxifloxacin and may be utilized for treatment in a multidrug regimen.

This study has certain limitations. Currently, molecular identification is used to identify related/newer NTM species. We could not perform molecular identification for our NTM isolates to show concordance. However, in resource limited settings where these newer technologies are not widely used either because of cost or lack of expertise, conventional test based NTM identification should be undertaken in an attempt to identify clinically significant isolates. Another limitation of this study is unavailability of clinical details for all the cases which limited our ability to assess their clinical significance.

## Conclusions

This is the first study reporting NTM species and their clinical significance isolated from clinical specimens from Pakistan. This study provides a baseline for future studies to compare NTM isolation and their antimicrobial susceptibilities.

## Abbreviations

NTM: Non-tuberculous mycobacteria; MOTT: Mycobacteria other than tuberculosis; HIV/AIDS: Human immuno-deficiency virus/acquired immuno-deficiency syndrome; DST: Drug susceptibility testing; MAC: *Mycobacterium avium* complex; RGM: Rapidly growing mycobacteria; SGM: Slow growing mycobacteria; JCIA: Joint commission international accreditation; LJ: Lowenstein-Jensen medium; MGIT: Mycobacteria growth indicator tube; AFB: Acid fast bacilli; PNB: Para-nitro benzoic acid; T2H: Thiophene-2-carboxylic acid hydrazide; ATCC: American type culture collection; CLSI: Clinical and laboratory standards institute.

## Competing interests

The authors declare that they have no competing interests.

## Authors’ contributions

IA participated in study design, collected strains, patients’ data, carried out the bench work, data analysis and drafting of manuscript. KJ conceived of the study, helped coordinating the study and manuscript. RH helped in study design, coordinated the study and reviewed the manuscript critically. All authors have read and approved the final manuscript.

## Authors’ information

RH (MBBS, PhD, FRCPath) is a Professor and consultant clinical microbiology at AKUH and Honorary Professor at the London School of Hygiene and Tropical Medicine, London, UK. She established Mycobacteriology laboratory services at AKUH, currently a candidate supranational laboratory for the Eastern Mediterranean Region of the World Health Organization.

KJ (MBBS, FCPS) is an Assistant Professor and consultant at AKUH. Her interests include Mycology as well as Mycobacteriology.

IA (MBBS, MSc) is currently studying for a fellowship degree in clinical microbiology of College of Physicians and Surgeons of Pakistan. He holds an MSc in Medical Microbiology from London School of Hygiene and Tropical Medicine, London, UK.

## Pre-publication history

The pre-publication history for this paper can be accessed here:

http://www.biomedcentral.com/1471-2334/13/493/prepub

## References

[B1] Karakousis PC Moore RD Chaisson RE Mycobacterium avium complex in patients with HIV infection in the era of highly active antiretroviral therapy Lancet Infect Dis 2004 13 9 557 565 15336223 10.1016/S1473-3099(04)01130-2

[B2] Kourbeti IS Maslow MJ Nontuberculous mycobacterial infections of the lung Curr Infect Dis Rep 2000 13 3 193 200 11095856 10.1007/s11908-000-0035-7

[B3] Henry MT Inamdar L O'Riordain D Schweiger M Watson JP Nontuberculous mycobacteria in non-HIV patients: epidemiology, treatment and response Eur Respir J 2004 13 5 741 746 15176690 10.1183/09031936.04.00114004

[B4] Piersimoni C Scarparo C Pulmonary infections associated with non-tuberculous mycobacteria in immunocompetent patients Lancet Infect Dis 2008 13 5 323 334 18471777 10.1016/S1473-3099(08)70100-2

[B5] Martin-Casabona N Bahrmand AR Bennedsen J Thomsen VO Curcio M Fauville-Dufaux M Feldman K Havelkova M Katila ML Koksalan K Non-tuberculous mycobacteria: patterns of isolation. A multi-country retrospective survey Int J Tuberc Lung Dis 2004 13 10 1186 1193 15527150

[B6] Falkinham JO Impact of human activities on the ecology of nontuberculous mycobacteria Future Microbiol 2010 13 6 951 960 20521938 10.2217/fmb.10.53

[B7] Jesudason MV Gladstone P Non tuberculous mycobacteria isolated from clinical specimens at a tertiary care hospital in South India Indian J Med Microbiol 2005 13 3 172 175 16100423 10.4103/0255-0857.16589

[B8] Ding LW Lai CC Lee LN Hsueh PR Disease caused by non-tuberculous mycobacteria in a university hospital in Taiwan, 1997–2003 Epidemiol Infect 2006 13 5 1060 1067 16492317 10.1017/S0950268805005698 PMC2870472

[B9] Griffith DE Aksamit T Brown-Elliott BA Catanzaro A Daley C Gordin F Holland SM Horsburgh R Huitt G Iademarco MF An official ATS/IDSA statement: diagnosis, treatment, and prevention of nontuberculous mycobacterial diseases Am J Respir Crit Care Med 2007 13 4 367 416 17277290 10.1164/rccm.200604-571ST

[B10] Clinical and Laboratory Standards Institute Susceptibility testing of Mycobacteria, Nocardiae and other aerobic Actinomycetes: Approved standard--second edition 2011 940 West Valley Road, Suite 1400, Wayne, Pennsylvania: Clinical and Laboratory Standards Institute 31339680

[B11] Brown-Elliott BA Nash KA Wallace RJ Jr Antimicrobial susceptibility testing, drug resistance mechanisms, and therapy of infections with nontuberculous mycobacteria Clin Microbiol Rev 2012 13 3 545 582 22763637 10.1128/CMR.05030-11 PMC3416486

[B12] World Health Organization Global tuberculosis control: epidemiology, strategy, financing: WHO report 2009 2009 Geneva: WHO 2009

[B13] Koneman EW Allen SD Janda WM Schereckenberger PC WJ W Color atlas and textbook of diagnostic microbiology 2005 Philadelphia: Lippincott 2005

[B14] Khanum T Rasool SA Ajaz M Khan AI Isolation-drug resistance profile and molecular characterization of indigenous typical and atypical mycobacteria Pak J Pharm Sci 2011 13 4 527 532 21959816

[B15] Hector JS Pang Y Mazurek GH Zhang Y Brown BA Wallace RJ Jr Large restriction fragment patterns of genomic Mycobacterium fortuitum DNA as strain-specific markers and their use in epidemiologic investigation of four nosocomial outbreaks J Clin Microbiol 1992 13 5 1250 1255 1583127 10.1128/jcm.30.5.1250-1255.1992 PMC265259

[B16] Safranek TJ Jarvis WR Carson LA Cusick LB Bland LA Swenson JM Silcox VA Mycobacterium chelonae wound infections after plastic surgery employing contaminated gentian violet skin-marking solution N Engl J Med 1987 13 4 197 201 3600710 10.1056/NEJM198707233170403

[B17] Maloney S Welbel S Daves B Adams K Becker S Bland L Arduino M Wallace R Jr Zhang Y Buck G Mycobacterium abscessus pseudoinfection traced to an automated endoscope washer: utility of epidemiologic and laboratory investigation J Infect Dis 1994 13 5 1166 1169 8169416 10.1093/infdis/169.5.1166

[B18] Arnow PM Bakir M Thompson K Bova JL Endemic contamination of clinical specimens by mycobacterium gordonae Clin Infect Dis 2000 13 2 472 476 10987707 10.1086/313940

[B19] Blossom DB Alelis KA Chang DC Flores AH Gill J Beall D Peterson AM Jensen B Noble-Wang J Williams M Pseudo-outbreak of mycobacterium abscessus infection caused by laboratory contamination Infect Control Hosp Epidemiol 2008 13 1 57 62 18171188 10.1086/524328

[B20] van Ingen J Boeree MJ van Soolingen D Mouton JW Resistance mechanisms and drug susceptibility testing of nontuberculous mycobacteria Drug Resist Updat 2012 13 3 149 161 22525524 10.1016/j.drup.2012.04.001

